# Highly diverse microbial community of regenerated seedlings reveals the high capacity of the bulb in lily, *Lilium brownii*

**DOI:** 10.3389/fmicb.2024.1387870

**Published:** 2024-06-05

**Authors:** Sauban Musa Jibril, Wu Yan, Yi Wang, Xishen Zhu, Zhou Yunying, Jie Wu, Ling Wang, Limin Zhang, Chengyun Li

**Affiliations:** ^1^State Key Laboratory for Conservation and Utilization of Bio-Resources in Yunnan, Yunnan Agricultural University, Kunming, China; ^2^Yunnan-CABI Joint Laboratory for Integrated Prevention and Control of Transboundary Pests, Yunnan Agricultural University, Kunming, China

**Keywords:** lily bulb, seedling regeneration, microbiome, vertical transmission, changes of microbial communities

## Abstract

Lily bulbs, which have both nutrient storage and reproductive functions, are a representative group of plants for studying the maintenance and transfer of plant-associated microbiomes. In this study, a comparison of the microbial composition of bulbs and their regenerated seedlings cultured under aseptic conditions, as well as subcultured seedlings that succeeded five times, was examined by amplicon sequencing. A total of 62 bacterial taxa and 56 fungal taxa were found to be transferred to the 5th generation in seedlings, which are the core microbiome of lily. After the regeneration of seedlings from bulbs, there was a significant increase in the number of detectable microbial species, and after 1, 3, and 5 successive generations, there was a decrease in the number of detectable species. Interestingly, some “new” microorganisms appeared in each generation of samples; for instance, 167 and 168 bacterial operational taxonomic units (OTUs) in the 3rd and 5th generations of seedlings that were not detected in either bulbs or seedlings of the previous two generations. These results suggest that bulbs can maintain a high diversity of microorganisms, including some with ultra-low abundance, and have a high transfer capacity to tuck shoots through continuous subculture. The diversity and maintenance of the microbiome can provide the necessary microbial reservoir support for regenerating seedlings. This habit of maintaining low abundance and high diversity may be biologically and ecologically critical for maintaining microbiome stability and function due to the sequestration nature of the plant.

## Introduction

Plants harbor diverse microorganisms such as archaea, bacteria, fungi, oomycetes, and viruses, collectively termed the plant microbiome that form a complex interaction that impacts plant health and productivity (Vorholt, 2012; Trivedi et al., 2020). The microbiome is necessary for plant growth and function and plays an essential role in nutrient availability acquisition and uptake, promoting biotic and abiotic stress, physiology regulation through microbe-to-plant signals, resistance to pathogens, and growth regulation via the production of phytohormones (De Santi Ferrara et al., 2012; Trivedi et al., 2020; Lyu et al., 2021; Ahmed et al., 2022). The soil is widely considered the origin and main source of plant microorganisms and is shaped by both soil type and host differences through the rhizosphere (Bulgarelli et al., 2013; Edwards et al., 2015; Gopal and Gupta, 2016). To recruit microorganisms, plants discharge exudates containing plant-derived signals, such as organic acids and sugars; plant-associated microbiota perceive the exudates signal by the use of chemotaxis and move towards the plant primarily through the use of flagella to initiate colonization (Trivedi et al., 2020). Plant microbiomes may vary between different plant species or cultivars, compartments, geographic locations, and developmental stages (Hamonts et al., 2018).

The plant-associated microbiota colonizes the rhizosphere (i.e., the zone of soil directly influenced by root exudation) and adheres to the root or leaf surface (rhizoplane and phylloplane) or the interior of roots and leaves (endosphere) (Martin et al., 2017). Plant endophytes are mainly bacteria and fungi and are present in plant tissues of all species without causing disease (Gouda et al., 2016). Several vital activities of the host plant are known to be influenced by the presence of endophytes. Endophytes can promote plant growth; fitness elicits a defense response against pathogen attack through the production of enzymes and secondary metabolites and improves nutrient uptake (Mejía et al., 2014; Bacon and White, 2016; Afzal et al., 2019).

Plants and associated microbiota form holobiont, and the evolutionary selection of holobiont likely occurs between the host and the microbes but also among microbes, and in many cases, the number of symbiotic microorganisms and their combined genetic information far exceeds that of their host (Rosenberg and Zilber-Rosenberg, 2014; Rosenberg and Zilber-Rosenberg, 2016). One of the key concepts of hologenome is that the microbiota, with its microbiome together with the host genome, can be transmitted from one generation to the next with fidelity and thus propagate the unique properties of the holobiont and the species (Rosenberg et al., 2007; Zilber-Rosenberg and Rosenberg, 2008). Previous studies reported that the similarities in the microbial composition between plants of the same species are mainly due to recruitment from the environment and are controlled by the plant genetics, morphology, and physiology (Bouffaud et al., 2014; Yeoh et al., 2017; Ahmed et al., 2024). Outside of a controlled environment (e.g., tissue culture condition), plants can recruit beneficial and non-beneficial microorganisms from varying sources available. Plant-associated microbiomes are either acquired from the environment through longitudinal transmission or vertically transmitted from parents to offspring. Previous studies have demonstrated the transmission of microorganisms from seed to seed in rice (Kim et al., 2022), bacterial vertical transmission in potatoes (Buchholz et al., 2019), and transmission of seeds beneficial endophytes in tomato (Bergna et al., 2018), and *Setaria viridis* (Rodríguez et al., 2020).

Lily, a perennial ornamental crop belonging to the family Liliaceae, has great ornamental, medicinal, and edible value (Rong et al., 2011). The plant belongs to the genus *Lilium* and is mostly grown from bulbs (Khan et al., 2008). China is known as an important distribution center for the genus *Lilium*, with 55 species having been reported in the country so far (Du et al., 2014). The *Lilium* spp. are of much significance as ornamental flowers but also have the potential to treat various diseases, especially anti-inflammatory and infectious diseases; the bulbs are traditionally used in medicine and are also an important source of food (You et al., 2010; Zhou et al., 2021). The bulbs have been reported to act as the food reserve for lilies (Lazare et al., 2019) and have a reproductive function. In addition, studies have shown that bulbs contain plant-growth endophytic microbes (Khan et al., 2020a,b; Khan et al., 2021). Despite its reproductive function, the transmission of lily bulbs associated with endophytic bacterial and fungal communities to developing seedlings and changes across generations have not been elucidated yet. This study aimed to assess the microbial composition and diversity of lily bulbs, their vertical transmission to developing seedlings under sterile tissue culture conditions, and the microbial succession and changes across generations. We hypothesized that the lily bulb harbors a diverse microbiome, some in ultra-low abundance, and regeneration of the bulb into seedlings and subculture leads to a more detectable and diverse microbiome that primarily originated from the bulb and vertically transmitted to the seedlings. Our findings will improve our knowledge on how the lily bulbs associated endophytic microbiomes are transmitted into young seedlings and may help in the development of sustainable strategies for improving the production of the plant.

## Materials and methods

### Experimental design, sample collection, explants preparation

Experiments were designed to study the lily bulb microbiome and its ability to transfer microbiome across seedling generations under tissue culture conditions (Supplementary Figure S1). The bulbs of lily (*Lilium brownii*) were collected from Sicheng, a lily planting base in Xundian County (103° 13′-103 ° 16′E), Kunming Yunnan province, China. The samples were placed in sterile plastic bags and immediately transported to the laboratory of the National Engineering Center of Yunnan Agricultural University. The mud and outer layer of the bulbs were peeled off to obtain the inner scales and washed under running tap water for 2 h. Furthermore, the scales of the lily bulb were soaked in 70% alcohol (v/v) for 30 s, followed by 0.1% mercuric chloride solution for 8 min, and finally rinsed with sterile distilled water 8 times. The sterilized explants were cut into approximately 1.5 cm × 1.5 cm, kept in sterile tubes, and cryopreserved at −80°C temperature until DNA extraction.

### Explant regeneration, preparation of culture medium, growth condition, and seedling subculture

For callus induction, the sterilized bulbs of lily (parent material) were induced in Murashige and Skoog (MS). 1/2 MS medium was used for explant germination and was prepared by melting 39.45 g/L in a hot plate and supplemented with 2.0 mg/L 2,4-dichlorophenoxyacetic acid, 0.5 mg/L naphthalene acetic acid (NAA), and 0.5 mg/L kinetin (KT) plant growth regulators for shoot induction, and the pH was adjusted to 5.8 using 1 M NaOH solution. The medium was dispensed into sterilized bottles, followed by autoclaving at 121°C for 20 min, and finally cooled in the laminar airflow cabinet (Kumar and Loh, 2012). Four explants were aseptically placed in each bottle and grown for 45 days (zero-generation seedlings). The cultures were maintained in a tissue culture room under a 16-h photoperiod with light intensity of 100 μmol m − 2 s − 1 at a temperature of 25 ± 2°C.

Seedling obtained from the initial propagation was subcultured into a new MS medium supplemented with plant growth regulators (NAA 1 mL/L + 6-BA 2 mL/L). After every 35 days, newly adventitious buds were subcultured into a new MS medium representing a new generation (first to fifth generations).

### DNA extraction

The microbial DNA extraction was conducted on an ultra-clean workbench, and all experimental equipment was sterilized to avoid contamination. Approximately 1.5 cm × 1.5 cm size bulbs and leaves of propagated seedlings were used with 15 replicates each. The samples were cut into pieces and packed into 2-ml centrifuge tubes; approximately 5 sterile zirconia beads of 3 mm were added to each tube and centrifuged thoroughly on the tissue grinder (Bertin Precellys Evolution, France). The DNA extraction was conducted using a DNeasy PowerSoil Kit (Qiagen, Germany) according to the manufacturer’s instructions. The DNA quality was investigated by running the extracted DNA on a 1.0% agarose gel electrophoresis.

### PCR amplification and sequencing

For bacteria, the V4 variable region of the 16S rRNA gene was amplified using the primers 515F (5’-GTGCCAGCMGCCGCGGTAA-3′) and 806R (5′- GGACTACHVGGGTWTCTAAT-3′). For fungi, the internal transcribed spacer II (ITS2) was amplified using primer pairs (GCATCGATGAAGAACGCAGC) and (TCCTCCGCTTATTGATATGC). All PCRs were carried out in 30 μL reactions with 15 μL Phusion^®^ High-Fidelity PCR Master Mix with GC Buffer from (New England Biolabs). The PCR conditions were as follows: 10 × Easy Taq Buffer 3 μL, dNTPs (2.5 mM) 2.4 μL, forward and reverse primers (10 μM) 1.2 each μl, Easy Taq Polymerase 0.3 μL, template DNA 10 ng, and ddH2O to make up to 30 μL. The PCR amplification program was as follows: pre-denaturation at 95°C for 5 min, followed by 35 cycles (denaturation at 95°C for 45 s, retreat at 55°C for 45 s, and extension at 72°C for 90s). Then, it was stably extended at 72°C for 7 min and finally stored at 4°C (PCR machine: Bio-Rad C1000 Touch TM). The PCR product is at −20°C until use. The PCR products were detected by 2% electrophoresis on agarose gel; the qualified PCR products were purified by magnetic beads and quantified by enzyme labeling, and the samples were mixed in equimolar concentrations according to the concentration of PCR products. Sequence libraries were generated using an Illumina TruSeq^®^ DNA PCR-Free Library Preparation Kit. The constructed library was quantified by Qubit and Q-PCR and sequenced on the NovaSeq6000 (Illumina). The sequencing was performed at Novogene Co. Ltd., Beijing, China.

### Gene amplicon sequence processing

The sequence reads were imported into QIIME2 (2022.8) for further processing and analysis (Bolyen et al., 2019). Samples were demultiplexed and sorted out based on their barcode and primer, and the DADA2 plugin was then used for quality filtering (Callahan et al., 2016). High-quality sequences were clustered into operational taxonomic units (OTUs) using an open reference vsearch algorithm in QIIME2 against 97% Silva OTU representative sequence database (v132, 2018) (Quast et al., 2012). Bacterial sequences were checked for chimeric sequence removal using the vsearch uchime-denovo algorithm (Edgar et al., 2011). Finally, the taxonomy assignment of non-chimeric sequences was assigned using the Naïve Bayes algorithm against the Silva database pre-trained classifier for the V4 region of the 16S rRNA regions in QIIME2. For the fungal ITS region, the taxonomic assignment was carried out using the UNITE database in QIIME2 (Nilsson et al., 2019). Fungal OTUs derived from plants and bacterial OTUs assigned to mitochondria and chloroplasts were removed using the QIIME taxa filter table.

### Data analysis

Phyloseq package (Mcmurdie and Holmes, 2013) in R statistical software was used to calculate the alpha diversity indices (Chao 1 and Shannon), and the results of alpha diversity for bacterial and fungal communities were visualized in boxplots using the R package “ggplot. Principal coordinate analysis (PCOA) was calculated based on Bray–Curtis distance using the vegdist function of the Vegan package (v3.5.0). The Kruskal–Wallis test in SPSS was used to calculate the significant differences among alpha diversity of microbial communities and was considered significant when *p* < 0.05. Community structure differences were calculated using permutational multivariate analysis of variance (PERMANOVA) with adonis function from the vegan package in R (Dai et al., 2023). Microbial co-occurrence network analysis was conducted using Sparse Correlations for Compositional data algorithm (SparCC) in R for OTUs at the genus level (*p* > 0.05 and correlation coefficient > 0.3) for bacterial and fungal communities (Friedman and Alm, 2012). Network visualization was conducted with Gephi (v0.9.2) using Fruchterman–Reingold (Bastian et al., 2009). Hub taxa of each network were defined as the top 10 taxa belonging to top degree and betweenness centrality (Gao et al., 2021). Chord diagrams were produced to visualize the interkingdom correlation at the phylum level using the circlize package in R (Gu et al., 2014). The phylogenetic tree of core taxa was constructed on MEGA (7.0) based on maximum likelihood (ML) with 1,000 bootstraps (Kumar et al., 1994); Evolview was used to beautify the phylogenetic tree (Zhang et al., 2012). All figures were later modified and combined with Adobe Illustrator 2019.

## Results

### Sequence summary and distribution of OTUs of lily bulb and regenerated seedlings

We explored the microbial communities associated with lily bulbs and tissue culture seedlings induced from the bulbs. A total of 6,074,394 bacterial V4 16S rRNA and 6,373,49 fungal ITS1 high-quality merged reads were obtained from 75 samples with an average read of 85,436 bacterial and 84,980 fungal reads per sample after quality control and removal of chimeras. The reads were clustered into operational taxonomic units (OTUs) at 97% sequence similarities. The distribution of bacterial OTUs is lower in the bulb (951 OTUs) compared with zero (1833 OTUs), first (1,245 OTUs), third (1,170 OTUs), and first-generation (1,156 OTUs), respectively. The number of fungal OTUs varies from 514 OTUs in the bulb, 1,030 OTUs in zero seedlings, and 750, 694, and 676 OTUs for the first, third, and fifth generations, respectively.

### Variation of bacterial and fungal communities of lily bulbs and seedlings

**Figure 1:**
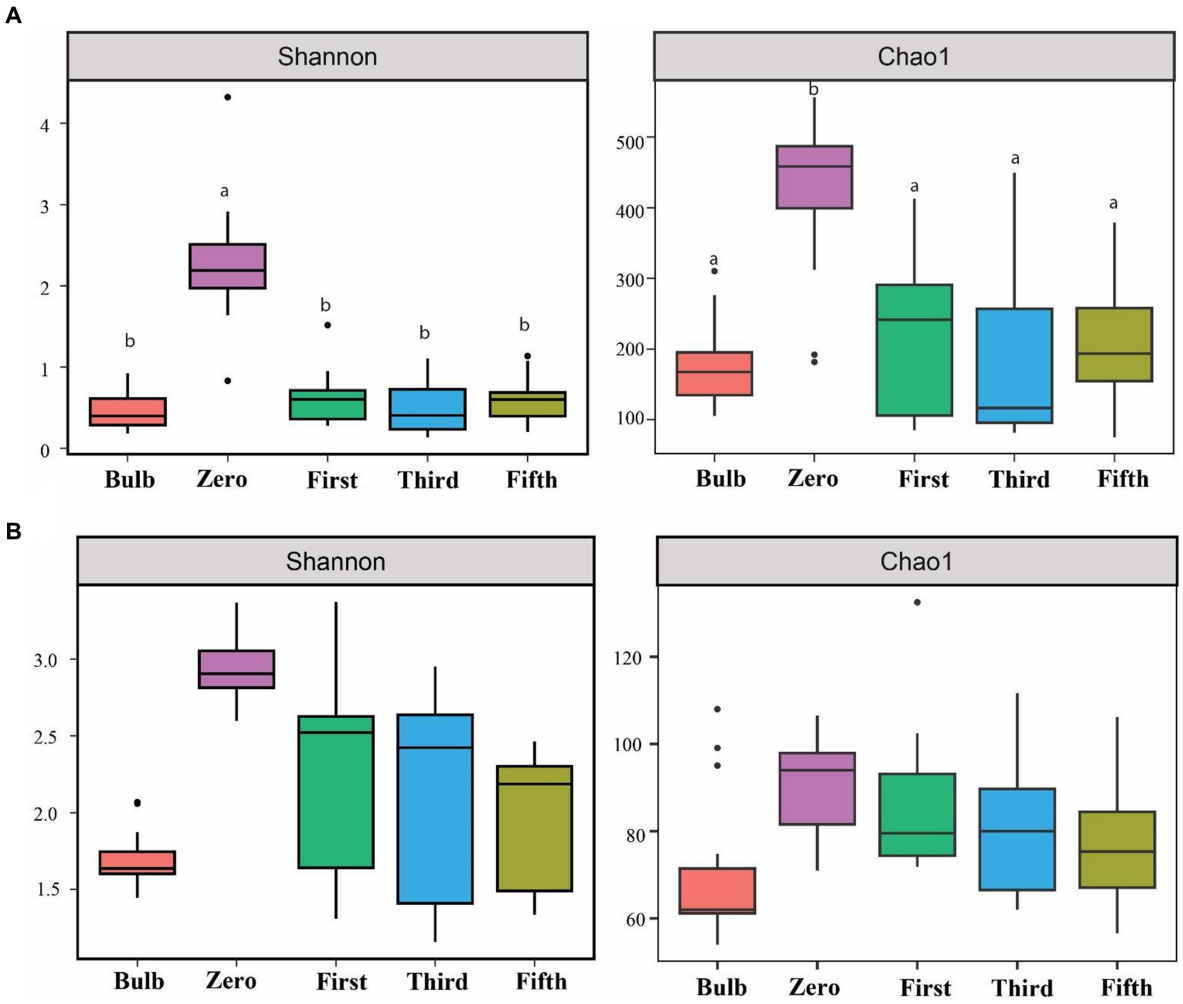
Assessment of alpha diversity across bulb and their regenerated seedlings using Shannon and Chao1 indices. **(A)** Bacterial and **(B)** Fungal community. Different lowercase letters indicate statistically significant according to the non-parametric Kruskal–Wallis test (*p* < 0.05).

**Figure 2:**
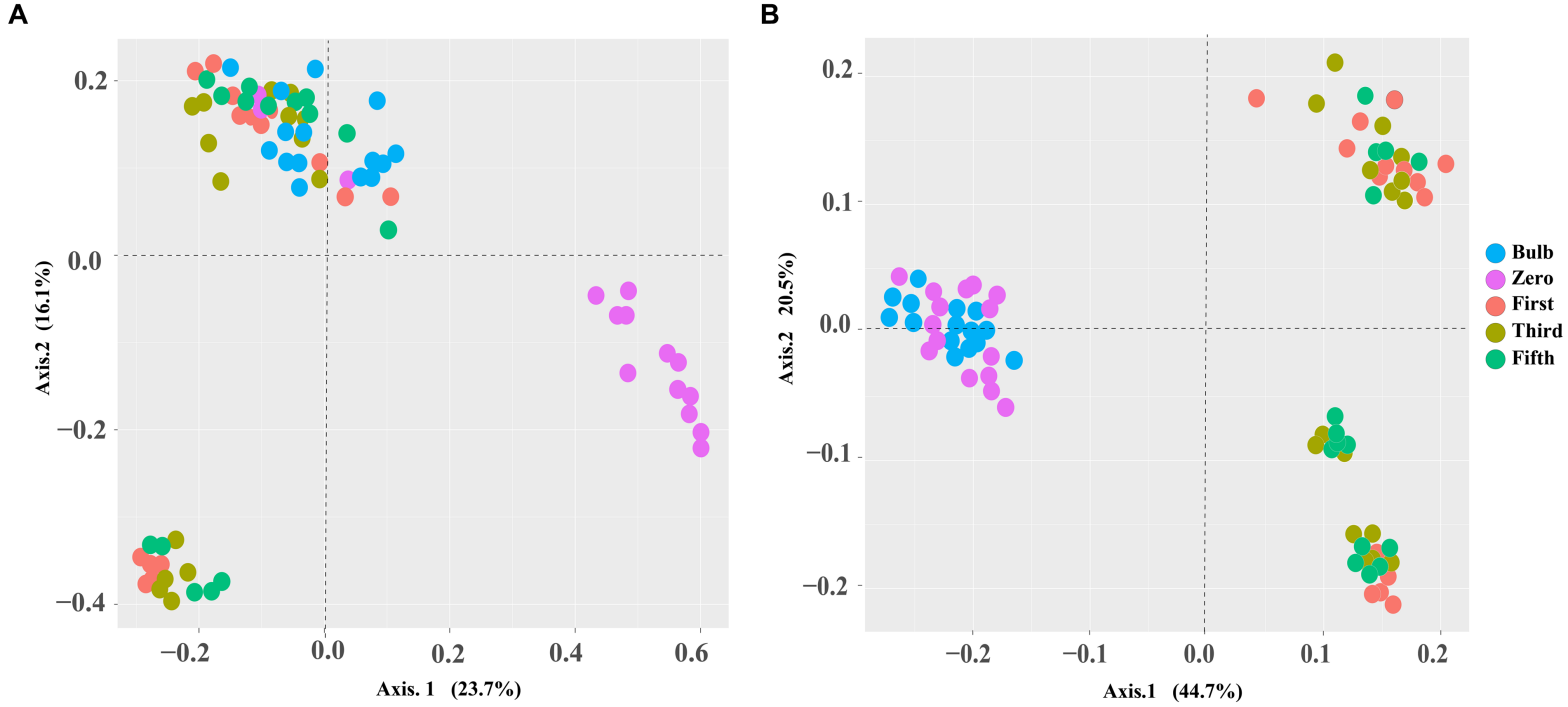
PCoA based on Bray–Curtis distance metrics demonstrating a pattern of bacterial **(A)** and fungal **(B)** communities of zero-, first-, third-, and fifth-generation bulbs.

For this analysis, we assessed the alpha and beta diversity of bacterial and fungal communities of bulb and seedling grown in an axenic MS medium. The Shannon and Chao1 indices determined alpha diversity. As shown in Figure 1, both bacterial and fungal α-diversity was significantly lower in the bulb than in the seedlings (*p* < 0.05), as indicated by Shannon and Chao1 indices. Zero generation showed significantly higher indices than first-, third-, and fifth-generation seedlings, which showed a gradual decline of diversity as the seedlings were further subcultured (Figures 1A,B). Moreover, principal coordinate analysis (PCOA) based on Bray–Curtis dissimilarity further showed the variation of bacterial and fungal communities. In the bacterial community, zero generation was grouped differently from the bulb, first-, third-, and fifth-generation seedlings (Figure 2A). In the fungal community, bulbs and zero generation were clearly separated from other seedlings (Figure 2B). The compositional variation was further confirmed by permutational multivariate analysis of variance (PERMANOVA) (bacterial: R^2^ = 0.50394, *p* = 0.001, fungi: R^2^ = 0.32988, *p* = 0.01) (Supplementary Table S1). Pairwise comparison indicated statistically significant variation in the bacterial (Supplementary Table S2) and fungal (Supplementary Table S3) communities (*p* < 0.05).

### Microbial community composition of lily bulbs

**Figure 3:**
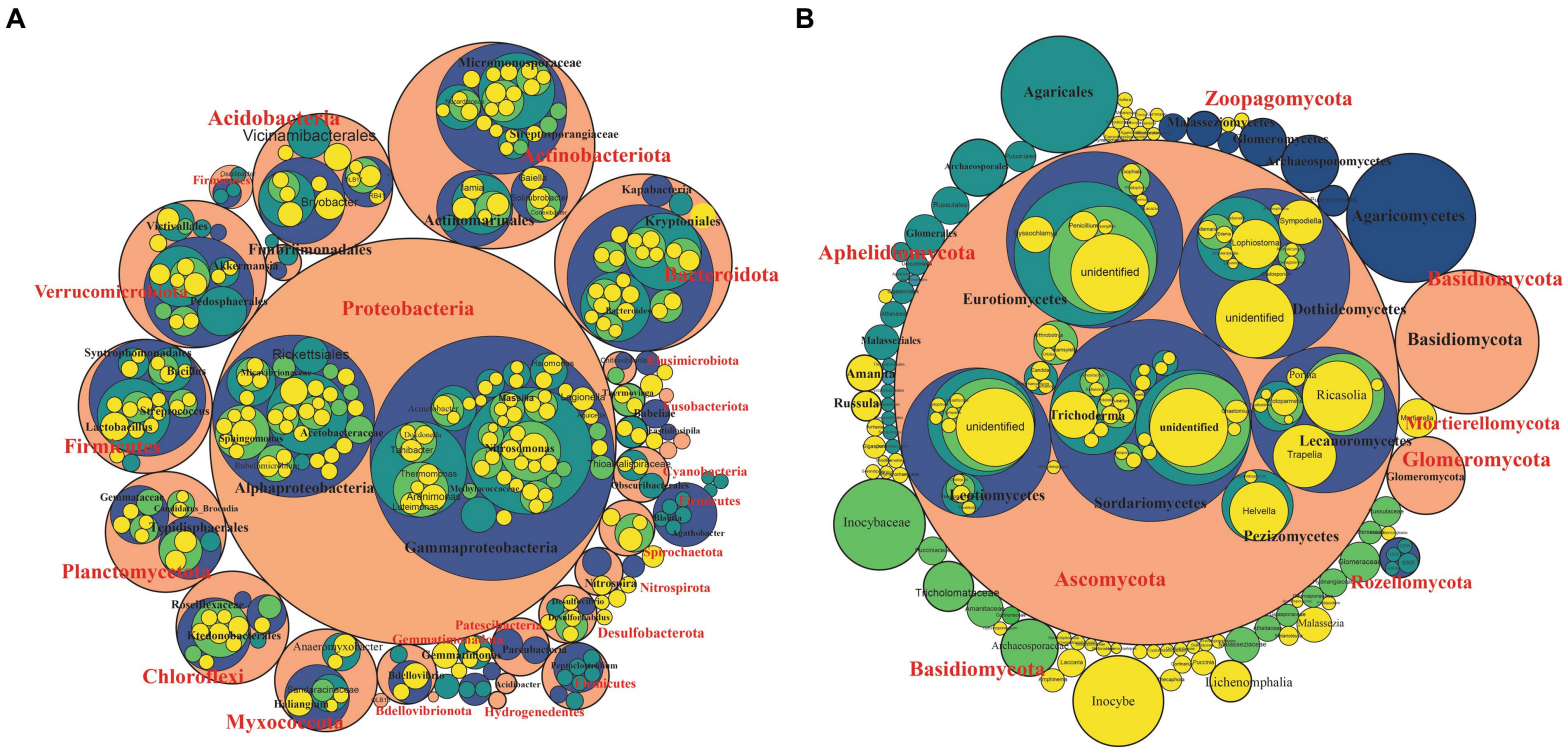
Phylum- to genus**-**level microbial community composition of the lily bulb. **(A)** Bacterial and **(B)** fungal community composition. The size of the circles represents relative abundance, colors showed different taxonomic levels. Pink = phylum, blue = class, green = order, light green = family, and yellow = species.

**Figure 4:**
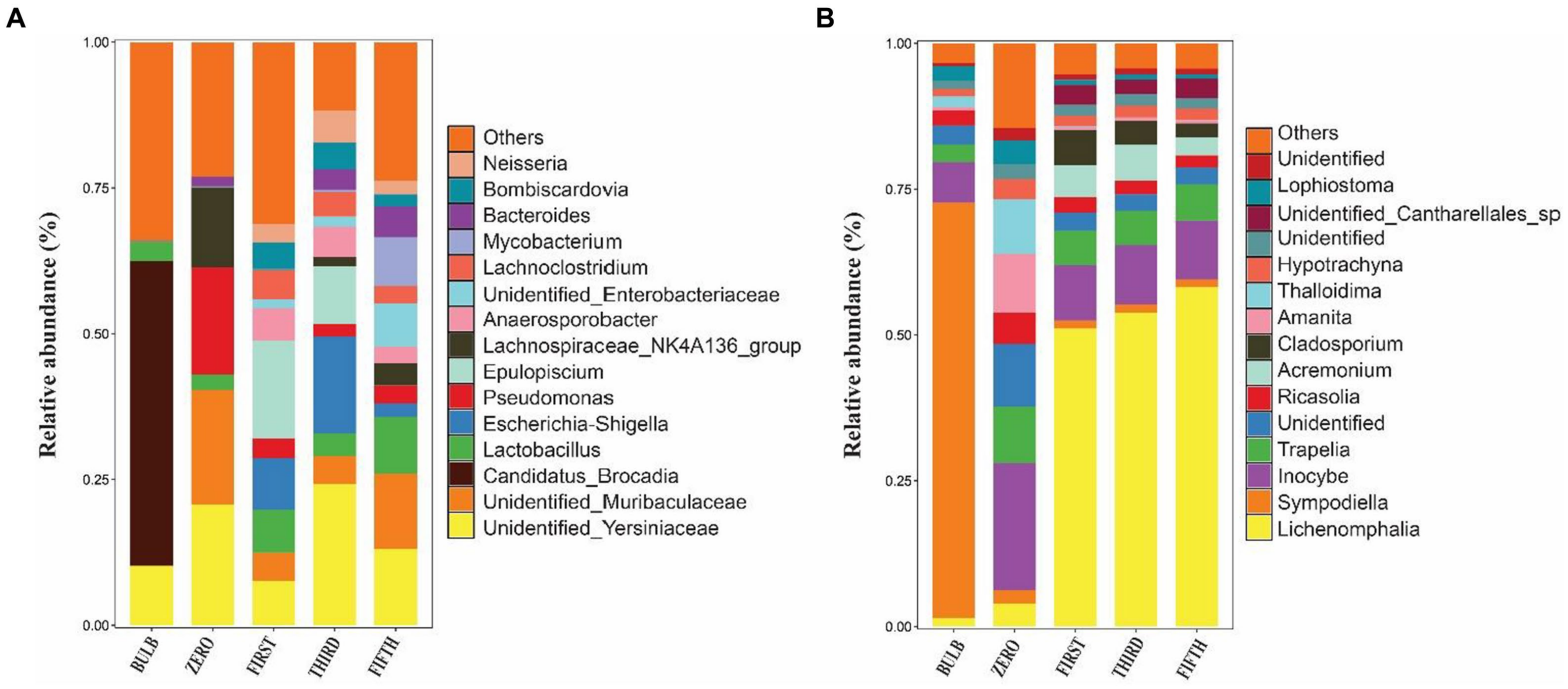
Relative abundance bar plots of the top 15 bacterial **(A)** and fungal **(B)** genera of bulb and regenerated seedlings.

**Figure 5:**
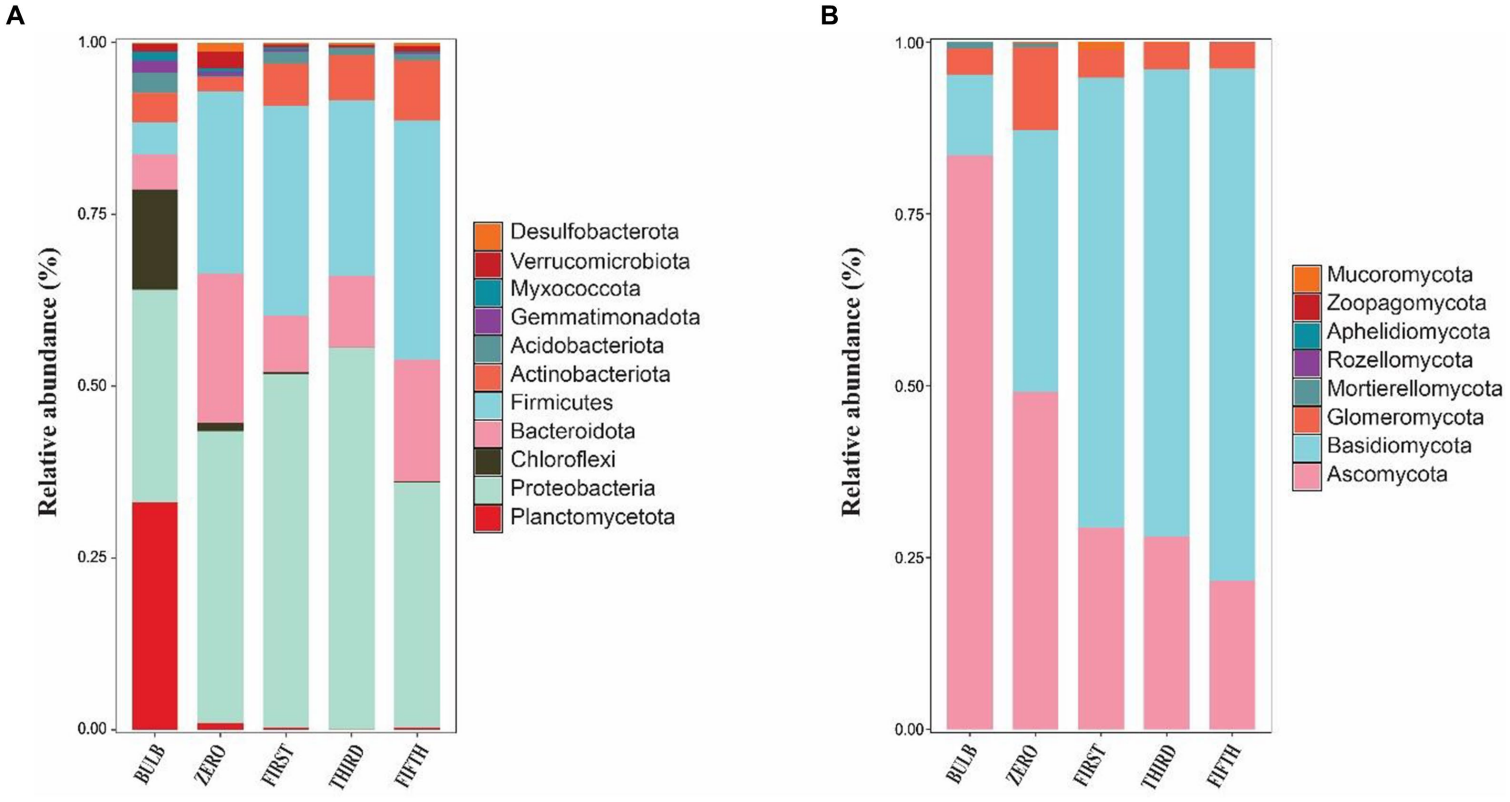
Phylum-level microbial community in bulb and their regenerated seedlings. **(A)** Relative abundance of bacterial and **(B)** fungal phyla. Only phylum with a relative abundance of 1% are represented.

We investigated the bacterial and fungal taxonomic composition of the bulb at the phylum and genera levels to assess the microbial composition before regeneration and propagation of the seedlings. The taxonomic annotation of bacterial and fungal OTUs showed that 951 and 514 OTUs were assigned to 30 bacterial and 7 fungal phyla, respectively. In the bacterial community, Planctomycetota (31.52%), Proteobacteria (29.42%), Chloroflexi (13.93%), Bacteroidota (4.86%), Firmicutes (4.39%), Actinobacteriota (4.07%), Acidobacteriota (2.85%), Gemmatimonadota (1.61%), Myxococcota (1.30%) and Verrucomicrobiota (1.03%) were the most abundant with relative abundance >1% (Figure 3A). At the genus level, *Candidatus_Brocadia* (28.97%), an unidentified genus belonging to *Yersiniaceae* (5.65%), and *Lactobacillus* (1.82%) were the most abundant genera (Figure 4A). In the fungal community, the most abundant phyla are Ascomycota (82.40%), Basidiomycota (11.64%), Glomeromycota (3.79%), Mortierellomycota, Aphelidiomycota, Rozellomycota, and Zoopagomycota (Figures 3B, 5B). At the genus level, *Sympodiella* (65.92%), *Inocybe* (6.50%)*, Trapelia* (2.69%), and *Lophiostoma* (2.67%) dominated the bulb fungal community (Figure 4B).

### Bulbs are capable of preserving a wide range of microbial communities in ultra-low abundance

We investigated and compared the microbial community of bulbs and their regenerated and subculture seedlings grown in an axenic MS medium free from other external sources of microorganisms, suggesting that the microbial communities of seedling originated from the bulb. We observed a significant increase in the number of detectable species in both bacterial (Supplementary Figures S2A–D) and fungal communities (Supplementary Figures S3A–D). The taxonomic bacterial and fungal composition differed between the bulb and their seedling. At the phylum level, the bacterial communities were dominated by Proteobacteria, Firmicutes, Bacteroidota, Actinobacteriota, and Acidobacteria (Figure 5A). Similarly, the predominant fungal phyla in the seedlings were Basidiomycota, Ascomycota, and Glomeromycota (Figure 5B). At the genus level, the bacterial community of zero-generation seedlings was dominated by unidentified bacteria belonging to Yersiniaceae, *Muribaculaceae, Pseudomonas, Lactobacillus, Lachnospiraceae_NK4136_group*, and *Bacteroides*. Meanwhile, *Epulopiscium*, unidentified bacteria belonging to Yersiniaceae, *Escherichia-Shigella, Anaerosporobacter, Neisseria, Bombiscardovia*, and *Lachnoclostridium* were the most abundant genera in the first and third generations. Mycobacterium, Lactobacillus unidentified Yersiniaceae, unidentified_Enterobacteriaceae, *Muribaculaceae, Bacteroides, and Neisseria* dominated the abundance of genera in the fifth-generation seedlings (Figure 4A). The genus *Candidatus_Brocadia* was significantly higher in the bulb compared with their seedlings whereas *Muribaculaceae, Pseudomonas, and Lachnospiraceae_NK4136_group* were significantly greater in the zero generation. *Escherichia-Shigella* and *Anaerosporobacter* were newly detected genera in the first, third, and the fifth generations (Supplementary Figure S4) (Kruskal–Wallis test, *p* < 0.05).

In the fungal community, zero-generation seedling was dominated by the genus *Inocybe, Trapelia, unidentified fungi, Ricasolia, Amanita, Thalloidima, Hypotrachyna, Sympodiella*, and *Lophiostoma.* In contrast to the bacterial community, the fungal communities of first-, third-, and fifth-generation seedlings were dominated by genera *Lophiostoma* (an average of 50.75%), *Inocybe, Trapelia, Acremonium, Cladosporium, and unidentified_Cantharellales_sp* (Figure 4B). The detection of new genera in the seedlings indicated that the bulb of lily is composed of ultra-low abundance microbial community that become detectable at seedling stages. Among the fungal genera, *Sympodiella* was statistically significant in the bulb. *Trapelia, Thalloidima, Ricasolia*, and *Amanita* were significantly higher in the zero generation (Kruskal Wallis test, *p* < 0.05; Supplementary Figure S5).

### Changes in the microbial community composition

**Figure 6:**
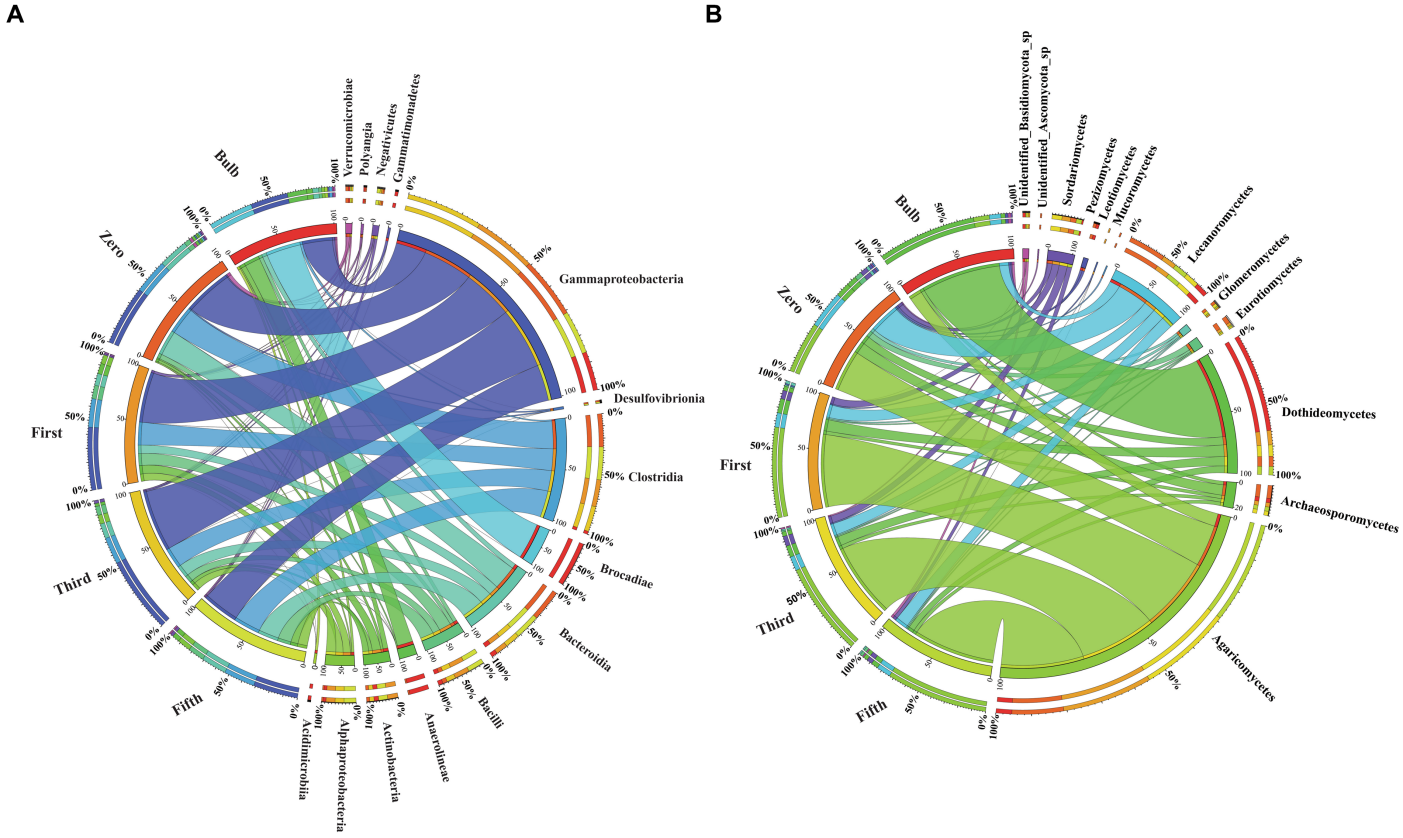
Changes in the bacterial and fungal microbial communities at the class level. **(A)** Bacterial **(B)** Fungal classes. Class with relative abundance >1% were visualized. The chord diagram visualization was executed using Circos software (Krzywinski et al., 2009). The change in the color size of the bands corresponds to the change members belonging to the class.

The taxonomic composition of bacterial and fungal communities changes between the bulb and their regenerated seedlings. We, therefore, further compare the community composition of the bulb and their regenerated seedlings at the class level. The bacterial community in the bulb is dominated by members of class *Brocadiae* (29.70%), *Gammaproteobacteria* (25.48%), and *Anaerolineae* (12.85%). In contrast, *Gammaproteobacteria, Clostridia*, and *Bacteroidia* were the most dominant classes in zero, first, third, and fifth generations (Figure 6A). Similarly, changes in the taxonomic composition were observed in the fungal community. In the bulb, the dominant class *Dothideomycetes* (66.83%), while the abundance of members belonging to this class decreased significantly after the regeneration of seedlings. *Agaricomycetes* and *Lecanoromycetes* were dominant in zero, first, third, and fifth generations (Figure 6B). The changes in the microbial community in bulbs and seedlings across generations suggest that the microbial community of the same host plant originating from the same source may vary from one generation to another.

### Bacterial and fungal taxa distribution among bulbs and their regenerated seedlings

**Figure 7:**
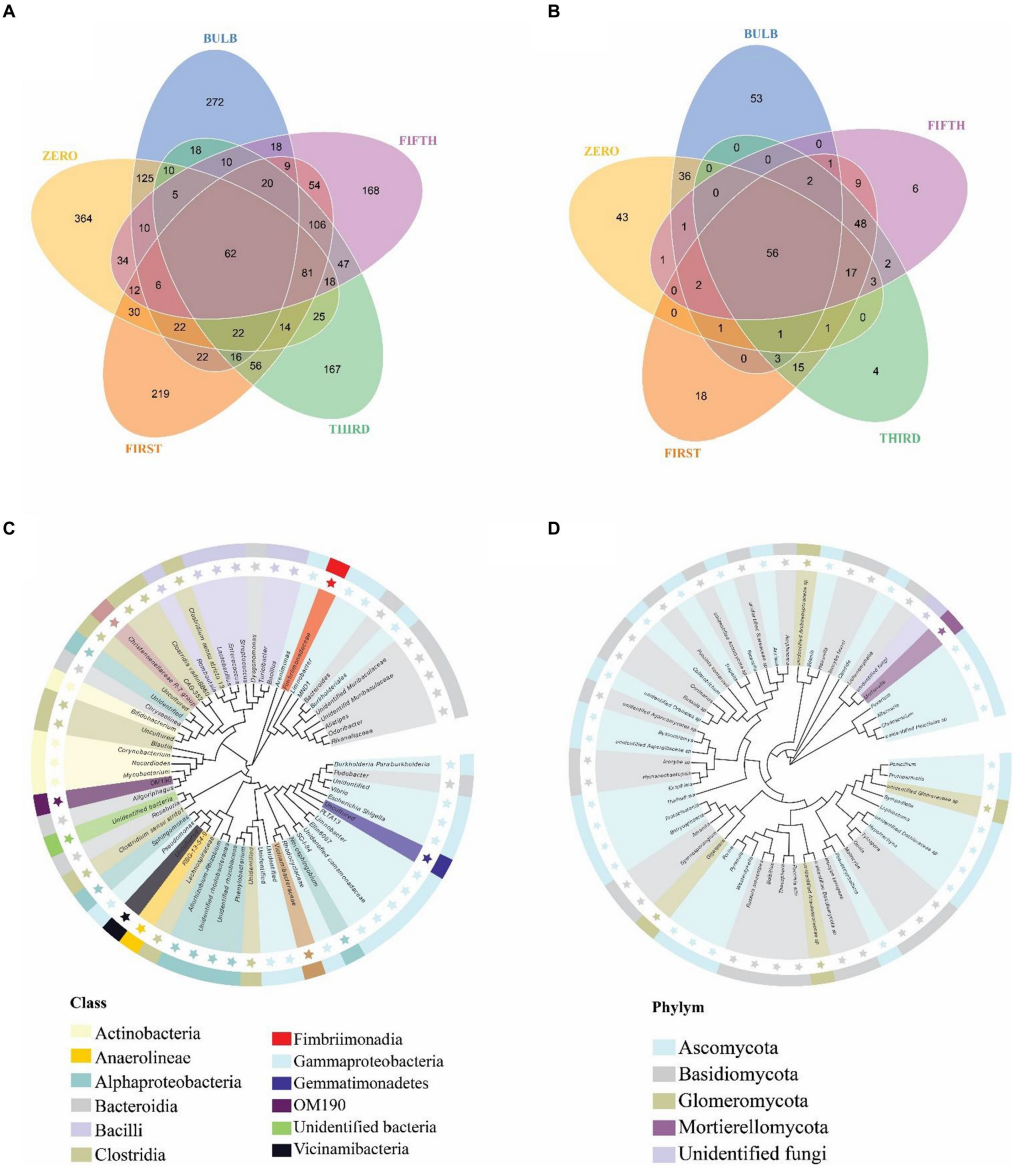
Distribution of bacterial and fungal taxa in bulb and seedlings. Venn diagram of bacterial **(A)** and fungal **(B)** taxa distribution. Phylogenetic tree of bacterial **(C)** and fungal **(D)** core taxa.

For this analysis, we classify microbiomes into transient, transmitted, and core microbiomes. We defined transient microbiome as the microbial community detected in the bulb or a seedling but not transmitted to another generation. The transmitted microbiomes are taxa that are found in bulbs and are vertically transmitted to the seedlings of different generations. The core microbiome is a set of microbial taxa that are conserved across all generations. Among the transient taxa, 272 bacterial and 53 fungal taxa were unique to the bulb, and 364 bacterial and 43 fungal taxa were unique to the zero regenerated seedlings. We found 125 bacterial and 36 fungal taxa that were shared between the bulb and the zero-generation seedlings (Figures 7A,B); 219 and 18 bacterial and fungal taxa were first-generation specific. Meanwhile, 167 and 168 unique bacterial taxa were found in third- and fifth-generation seedlings, respectively. Four and six unique taxa were detected in third- and fifth-generation seedlings in fungal communities. The transient bacterial community was dominated by members of Proteobacteria, Chloroflexi, Actinobacteria, and Bacteroidota, while fungal communities mainly belonged to Ascomycota and Basidiomycota (Supplementary Table S4).

We detected 62 bacterial and 56 fungal core taxa that were vertically transmitted and shared from bulbs to fifth-generation seedlings (Figures 7A,B) (Supplementary Table S5). The phylogenetic analysis of the core taxa showed a random phylogenetic distribution. The bacterial core taxa mainly belonged to class Gammaproteobacteria, Bacteroidia, Bacteroidia, Alphaproteobacteria, and Clostridia, whereas fungal core taxa were dominated by phylum Ascomycota and Basidiomycota (Figures 7C,D). The relative abundance of the core microbiome varies between the bulb and the seedlings. Among them, genera *Nocardioides, Vibrio, and Fimbriimonadaceae*, Unidentified genera belonging to the order Vicinamibacterales, *Elin6067* exhibited higher relative abundance in the bulb. Still, they showed low abundance in the seedlings, whereas genera like *Pseudomonas, Muribaculaceae_Bacteroideles, Unidentified_ Armatimonadota, Burkholderia-Caballeronia-Paraburkholderia, Alistipes*, Unidentified genera belonging to the family *Yersiniaceae* exhibited high abundance in the seedlings compared with the bulb (Supplementary Figure S6A). A similar pattern was observed in the fungal community composition (Supplementary Figure S6B).

### Characteristics of the bulb and regenerated seedlings microbiome co-occurrence network

**Figure 8:**
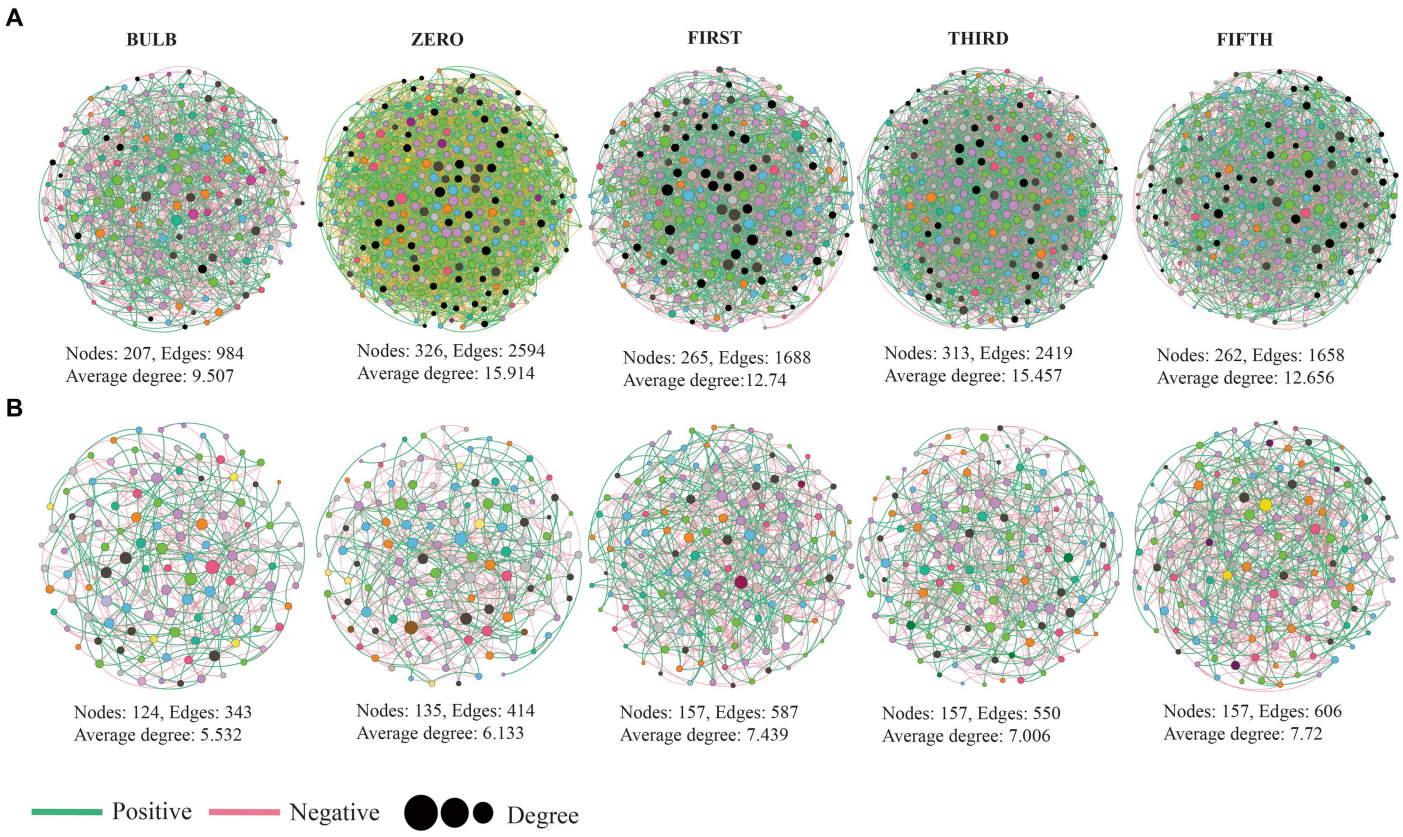
Microbial co-occurrence network analysis of lily bulb and seedlings at the genus level **(A)** bacterial **(B)** fungal networks. Each node represents microbial genus and different colors represent phylum and class level for bacterial and fungal networks, respectively. The size of the node indicated its degree of correlation. The green and red lines indicate positive and negative interactions, respectively.

To investigate the interaction of microbial communities, we constructed intra and inter-kingdom microbial co-occurrence networks at the genera level. The network complexity was defined based on the number of nodes, average degree, and number of edges. In the intra-kingdom network, higher network complexity was found in the bacterial network compared with fungi (Figure 8). We found a significant increase in bacterial network complexity in the zero generation and a gradual decrease from the first to fifth generation (Figure 8A). In contrast, the fungal networks showed similar patterns in topological characteristics in the first to fifth generations (Figure 8B) (Supplementary Table S6). The top ten nodes with the highest degree and betweenness centrality scores were assigned as hub nodes. In bacterial networks, hub nodes in the bulb include *Arenimonas, IMCC26256*, and *AKYH767*, as bacterial taxa such as *ASF356, Candidatus_Aquiluna*, and *Escherichia, while Shigella* was assigned as hub taxa in seedlings.

In the fungal network, hub taxa such as *Inocybe, Didymella*, and *unidentified* were detected in the bulb, whereas *Cladosporium, Trapelia*, and *Lasiodiplodia* were assigned in zero generation (Supplementary Table S7). Our results based on bacterial–fungal interkingdom co-occurrence networks showed similar patterns of complexity with bacterial intrakingdom networks (Supplementary Figure S7). The interkingdom network correlation was dominated by negative correlations (Supplementary Table S8). Hub taxa in the interkingdom network were assigned to both bacterial and fungal genera (Supplementary Table S9). The correlations between nodes were mostly between genera belonging to Proteobacteria, Firmicutes, Planctomycetota, Actinobacteria, Chloroflexi, Bacteroidota, and fungal phyla Ascomycota and Basidiomycota (Supplementary Figure S8).

## Discussion

Plant microbiome consists of diverse microbial communities; these microbes either live inside or outside of plant tissues and play a vital role in their fitness, health, growth, and development. In this study, we investigated lily bulb microbial composition, vertical transmission, and changes across seedling generations. We surface sterilized, extracted, and sequenced the bulb microbial community, and the sequencing results showed a highly diverse and distinct microbial community in the bulb. We then induced the bulb into seedlings and subcultured into different generations. Our results showed that bulbs of lily consist of diverse undetectable microbial communities that become detectable at the seedling stage. Our results demonstrated that the bulb of a lily is an important source of the microbiome that can be transmitted from one generation to another and contribute to the establishment of the lily microbial community.

### Lily bulb showed highly diverse and distinct microbial communities

Plant seeds are the primary source of microbial community for young developing seedlings, and they are essential for early development. The present study demonstrated that the bulb of a lily is composed of highly diverse bacterial and fungal communities. The highly diverse microbial community of lily bulbs might be partially inherited from the parent plant and soil. These results are in accordance with the previous studies on seed microbial communities (Wassermann et al., 2019; Abdelfattah et al., 2021).

The bulb bacterial communities were dominated by phylum Planctomycetota Proteobacteria, Chloroflexi, and commonly dominated by the genus *Candidatus_Brocadia, an* unidentified bacteria belonging to Yersiniaceae and *Denitratisoma*. Many previous studies reported *Candidatus_Brocadia* an anaerobic ammonium oxidation in plant roots that contributes to the denitrification in plants grown in in eutrophic wetlands and Chinese paddy soil (Bai et al., 2015; Zhang et al., 2021). These suggest that the genus might be horizontally acquired from the surrounding soil environment. Similarly, the fungal community was dominated by the genera *Sympodiella* and *Inocybe*. In contrast to bacterial communities, these genera were transmitted to all generations. These findings suggest that bulbs of lily could be a potential source of microbial community for developing seedlings.

### Lily bulbs can transmit highly diverse microbiomes through continuous subculture

The diversity of microbial communities differed between the bulb and their regenerated seedlings. Alpha diversity indices were significantly higher in the regenerated seedlings, indicating that highly diverse bulb bacterial and fungal could be vertically transmitted and become detectable at the seedling stage as the seedlings were induced and regenerated in tissue cultures conditions with no external source of microbes; we assume that all the seedling microbial communities originated from the bulb and transmitted to the growing seedlings. The increase in microbial diversity might be due to the physiological and biochemical changes from the bulb to the seedling stage. Morphological differences, physiological metabolism, and variation of metabolites could lead to niche differentiation between compartments during plant development (Kim and Lee, 2021). Moreover, the host developmental stage could also influence microbial diversity (Xiong et al., 2021). The higher abundance and differentiation of microbial community in oak seeds and seedlings were shown to be due to changes in physiological conditions and transmission from root to phyllosphere (Abdelfattah et al., 2021). Despite showing a significant increase at zero generation, microbial diversity showed a gradual decrease across generations. This might be due to the fact that the seedling was grown on a sterile medium without external microbial sources. This is in accordance with the previous results that seeds of plants grown over several generations in sterile substrate showed a decline in bacterial richness and seed vigor after generations further confirming a soil as a reservoir of seed microbial community (Rodríguez et al., 2020). In contrast to this study, we do not observe any visible phenotypic variations among the seedlings after being subcultured to fifth generations.

We found that the bacterial community of zero generation was dominated by Muribaculaceae*, an* unidentified genus belonging to Yersiniaceae, *Pseudomonas, Lachnospiraceae_NK4A136_group, Akkermansia, Luteibacter*, and *Burkholderia-Caballeronia-Paraburkholderia*. *Pseudomonas, Luteibacter*, and *Burkholderia-Caballeronia-Paraburkholderia* related species are well-known plant and human pathogens; they also promote plant growth and rhizosphere competence and help in providing special adaptability to plants (Compant et al., 2008; Li et al., 2019; Sah et al., 2021). Genera *Snodgrassella, Lactobacillus*, Saccharibacter*, Stenotrophomonas Anaerosporobacter, Lachnoclostridium, Pseudomonas, Stenotrophomonas, Gilliamella, Escherichia-Shigella, Bacillus, Bacteroides*, and *Mycobacterium* are the dominant genera in first to fifth generations. Plant-beneficial taxa belonging to *Bacillus, Pseudomonas*, and *Stenotrophomonas* have been using as plant growth promoters and biocontrol agents (Ryan et al., 2009; Saxena et al., 2020). *Mycobacterium* was also reported in promoting plant growth under stress conditions (Egamberdieva, 2012).

We found that community of zero-generation seedlings are dominated by genera such as *Sympodiella, Thalloidima, Lophiostoma, Ricasolia, Trapelia*, and *Tylospora*. Contrary to bacteria, the fungal community in the subsequent generations are dominated by *Lophiostoma*, *Inocybe, Acremonium*, and *Cladosporium*. This suggests that fungal community is more even and stable across the generation. Although the function of plant-associated fungi are less reported, *Cladosporium* has been reported as core taxa and also vertically transmitted from seed to leaves in forbs (Hodgson et al., 2014; Trivedi et al., 2020). These results suggest that the bulb of lily could transmits highly diverse microbial communities that become dateable at seedling stages.

### The distribution and changes of detectable taxa across generations

We found that the larger amount of bacterial and fungal communities detected at bulb were vertically transmitted to the zero-generation seedling. Previous study showed that substantial number of seed bacterial community are vertically transmitted to the next seed of generation (Hardoim et al., 2012; Kim and Lee, 2021). Although the number of vertically transmitted taxa declined across the generations (Figures 7A,B), we suggest that the decline of number of transmitted taxa might be due to the increase of host natural selection across generations. Previous study showed that the decrease in vertical transmission can increase microbiome variation and improve the change of offspring with right microbial combination (Bruijning et al., 2022).

We identified the existence of unique bacterial and fungal taxa in each seedling generation termed a transient microbiome, which we believed originated and transmitted from the bulbs. The transient microbial community belonged to members of Proteobacteria, Chloroflexi, Firmicutes, Actinobacteriota, Bacteroidota, Planctomycetota, Ascomycota, and Basidiomycota in lower abundance (Supplementary Table S4). Previous study reported transient taxa in seedlings grown in sterile substrates (Abdelfattah et al., 2021; Kim and Lee, 2021). We found 62 bacterial and 56 fungal core taxa that are conserved among the bulb and all generations. Core microbial communities are known to influence various plant functions, serving as microbial vehicles carrying replicators (genes) necessary for plant fitness (Lemanceau et al., 2017). Many of the members of the bacterial core taxa including *Sphingomonas, Pseudomonas, Bacteroides, Lactobacilli, Pseudomonas*, and *Bacillus* are well known in plant growth promotion and biocontrol of plant diseases (Asaf et al., 2020; Trivedi et al., 2020; Afanador-Barajas et al., 2021; Lidbury et al., 2021; Matsumoto et al., 2021). Previous research studies reported fungal core taxa *Gigaspora_margarita* and *Penicillium* as plant growth promoters, biocontrol, and biofertilizers (Khan et al., 2008; Radhakrishnan et al., 2014; Klinsukon et al., 2021). These results demonstrated that despite changes of microbial community across generation lily is capable of retaining and transmitting core bacterial and fungal taxa. Further research on the role of the core taxa will increase our understanding of their functional role in lily.

### Changes in microbial co-occurrence networks complexity

Microbial network analysis revealed the variation of microbial community interactions across the generations. The microbial interactions with one another play a crucial role in their functions (Sessitsch et al., 2019). Here, the results of co-occurrence network showed that different pattern across the generation, which could be due to the variation of niches (Ma et al., 2020). Comparison of topological characteristic between the networks was conducted to assess their complexity. The complexity of networks based on number of nodes, edges, and clustering coefficient was higher in seedlings than the bulb (Figure 7; Supplementary Figure S6). Clustering coefficient represents the complexity of the networks and strong interactions among microorganisms (Guo et al., 2022). According to the interactions of microbial communities, negative correlations predominate the interactions (Supplementary Tables S6, S8). Ecological modeling showed that more negative association between microbial communities promote their stability (Coyte et al., 2015). The frequency of positive interaction increases under drought stress (Gao et al., 2022).

Identifying hub nodes in the network could be used to determine their role in the microbial community. Nodes with higher interactions with others in the network are theoretically expected to play an important functional role than those with less connection (Shi et al., 2020). Based on their degree and betweenness centrality, top 10 genera were assigned as hub nodes (Supplementary Tables S7, S9). Different hub nodes were detected at each generation. It has been reported that variation of niches might result in different interaction and functional role of the same organisms (Liu et al., 2023). Some of the identified hub nodes such as *Trichoderma*, *Pseudoalteromonas, Filobasidium, Sphingomonas, Rhizobacterium, Exiguobacterium*, and *Desulfovibrio* have been reported as novel plant growth promoters through production of secondary metabolites, phytohormones, nitrogen fixation, and biological control (Dastager et al., 2010; Nieto-Jacobo et al., 2017; Asaf et al., 2020; Chen et al., 2020; Navarro-Torre et al., 2020; Mistry et al., 2022).

## Conclusion

The present study provides an insight into the microbial composition and diversity of lily bulb and their regenerated seedlings. Our results demonstrated that lily bulb does not only serve as storage and reproductive organ but also serves as a reservoir of microbial communities. Through comparison of microbial communities associated with the bulb and their regenerated seedlings, we demonstrated that the bulb can maintain highly abundance microbial communities in low abundance that are vertically transmitted and become detectable at seedling stages. The data suggested a clear evidence of changes in detectable microbial community across different plant generations. We also identified some bacterial and fungal taxa that are persistently inherited from one generation to another, suggesting that the host plant could select and conserve specific taxa. Moreover, it is important to further assess the changes of microbial communities of further generations in tissue culture and soil niches and their functional role in the plant. The overall findings suggest that lily bulb is a reservoir of microbial communities in low abundance that can be transmitted across seedling generations providing a young seedling with necessary microbial community. Our study provides a basis for understanding the relationship between lily bulb and seedling microbiome across generations.

## Data availability statement

The datasets presented in this study can be found in online repositories. The names of the repository/repositories and accession number(s) can be found in the article/Supplementary material.

## Author contributions

SJ: Software, Visualization, Writing – original draft, Writing – review & editing. WY: Methodology, Writing – original draft, Writing – review & editing. YW: Funding acquisition, Validation, Writing – original draft, Writing – review & editing. XZ: Validation, Writing – original draft, Writing – review & editing. ZY: Methodology, Writing – original draft, Writing – review & editing. LW: Methodology, Writing – original draft, Writing – review & editing. LZ: Methodology, Writing – original draft, Writing – review & editing. CL: Funding acquisition, Investigation, Resources, Supervision, Writing – original draft, Writing – review & editing. JW: Formal analysis, Writing – review & editing.

## Supplementary material

SUPPLEMENTARY FIGURE S1 Schematic diagram of experimental route followed in this research. Detailed description of the experiment was described in the materials and methods.

SUPPLEMENTARY FIGURE S2 Phylum to genus comparative analysis of the microbial composition among seedling generations. **(A–D)** zero, first, third and fifth-generation bacterial community compositions. Size of the circles represent their relative abundance and colors showed different taxonomic levels.

SUPPLEMENTARY FIGURE S3 Phylum to genus comparative analysis of the microbial composition among seedling generations. **(A–D)** zero, first, third and fifth-generation fungal community compositions. Size of the circles represent their relative abundance and colors showed different taxonomic levels.

SUPPLEMENTARY FIGURE S4 The box plots display the significant difference between between differentially abundant bacterial genera. The lowercase letters on each box plot display significant differences among samples (Kruskal wallis test, *p* < 0.05).

SUPPLEMENTARY FIGURE S5 The box plots showing the significant difference between differentially abundant fungal genera. The lowercase letters on each box plot display significant differences among samples (Kruskal wallis test, *p* < 0.05).

SUPPLEMENTARY FIGURE S6 Relative abundance of core taxa among different samples **(A)** Bacterial and **(B)** Fungal communities. Heat maps were generated using Bioinformatics.com.cn (https://bioinformatics.com.cn/plot_basic_cluster_heatmap_plot_024). The bluer the color, the higher the relative abundance.

SUPPLEMENTARY FIGURE S7 Inter-kingdom co-occurrence network analysis at genus level **(A)** Bulb **(B)** Zero generation **(C)** First, **(D)** Third, and **(E)** Fifth generations. Node size indicates the degree of correlation. Pink nodes showed bacterial genera, green indicate fungal genera. The hub nodes is represented by yellow nodes. The green and red lines indicate positive and negative interactions respectively.

SUPPLEMENTARY FIGURE S8 Chord plots illustrating the co-occurrence interactions between dominant taxa. Different color indicates different bacterial and fungal phyla.
