# Supplementary material for: Highly diverse microbial community of regenerated seedlings reveals the high capacity of the bulb in lily, Lilium brownii
Source: Front Microbiol. 2024 Jun 5;15:1387870. doi: 10.3389/fmicb.2024.1387870 (PMC11188333; doi:10.3389/fmicb.2024.1387870)
Supplement: Supplementary file 2 [file Data_Sheet_1.docx]

**High-diverse microbial community of regenerated seedlings reveals the high capacity of the bulb in Lily, *Lilium brownie***

Sauban Musa Jibril**^✝^** ^a,b,c^, Wu Yan**^✝^** ^a,b^, Yi Wang**^✝^** ^a,b^, Xishen Zhu ^a,b^, Zhou Yunyin ^a,b^, Jie Wu ^a,b^, Ling Wang ^a,b^, Limin Zhang ^a,b^, Chengyun Li ^a,b *^

^a^State Key Laboratory for Conservation and Utilization of Bio-Resources in Yunnan, Yunnan Agricultural University, Kunming, China.

^b^Yunnan-CABI Joint Laboratory for Integrated Prevention and Control of Transboundary Pests, Yunnan Agricultural University, Kunming, Yunnan 650201, China.

^c^Department of Biological Sciences, Al-Qalam University, 2137, Katsina, Nigeria.

*Correspondence: licheng_yun@163.com

**^✝^**These authors contributed equally to this work.

**Supplementary figures**

**
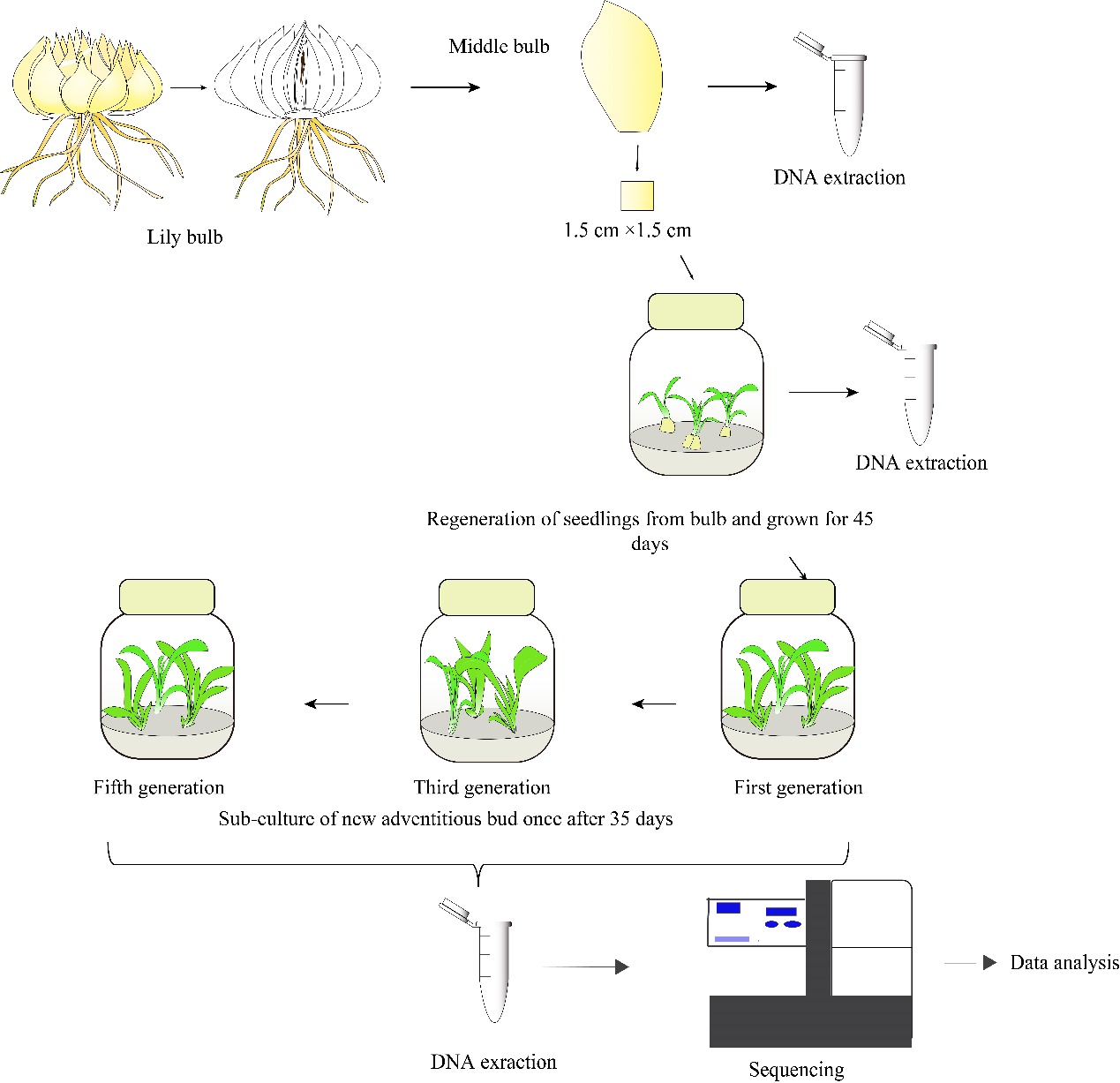
**

**Figure S1.** Schematic diagram of the experimental route followed in this research. Detailed description of the experiment was described in the materials and methods.


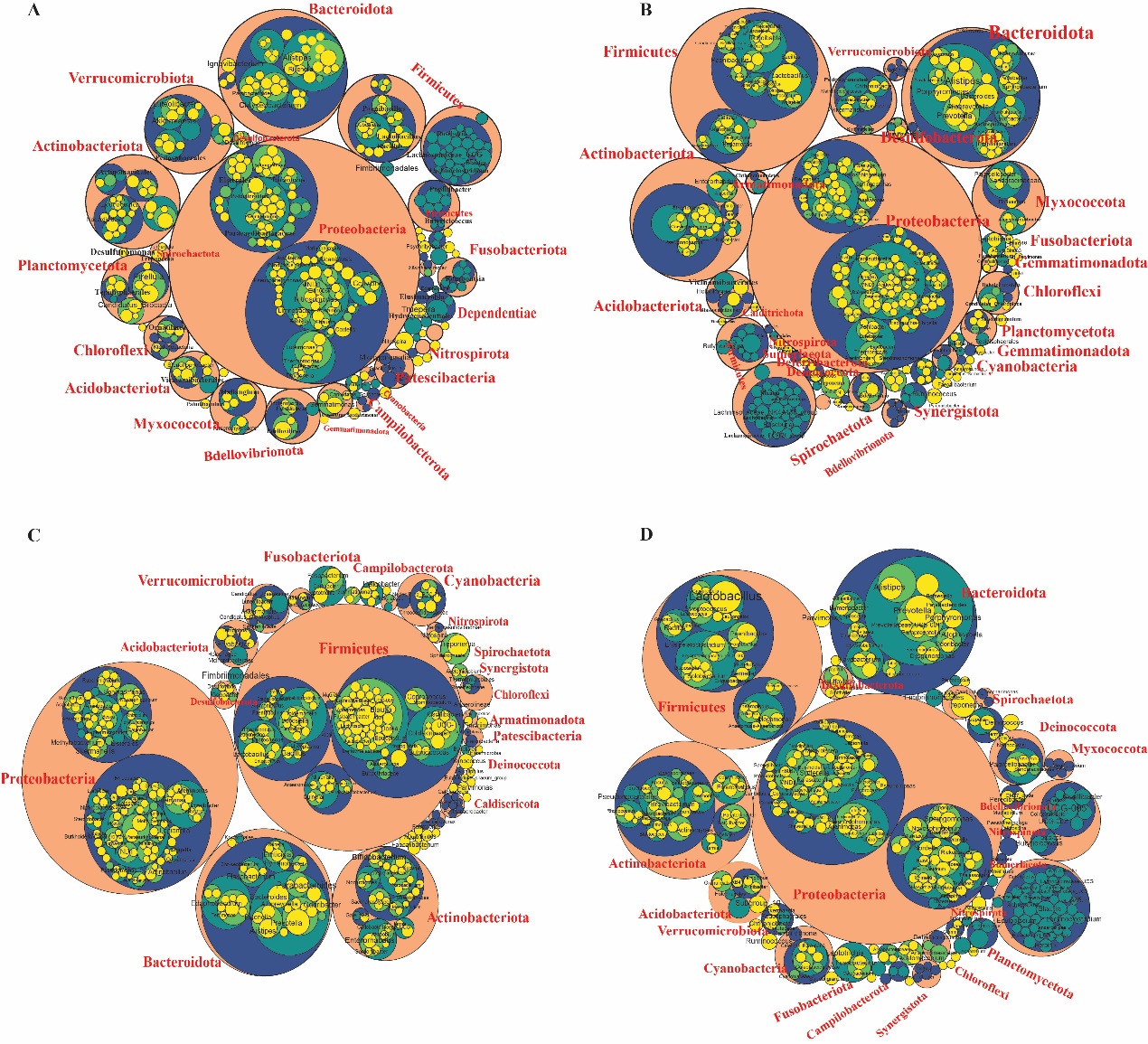


**Figure S2.** Phylum to genus comparative analysis of the microbial composition among seedling generations. **(A-D)** zero, first, third and fifth-generation bacterial community compositions. Size of the circles represent their relative abundance and colors showed different taxonomic levels.


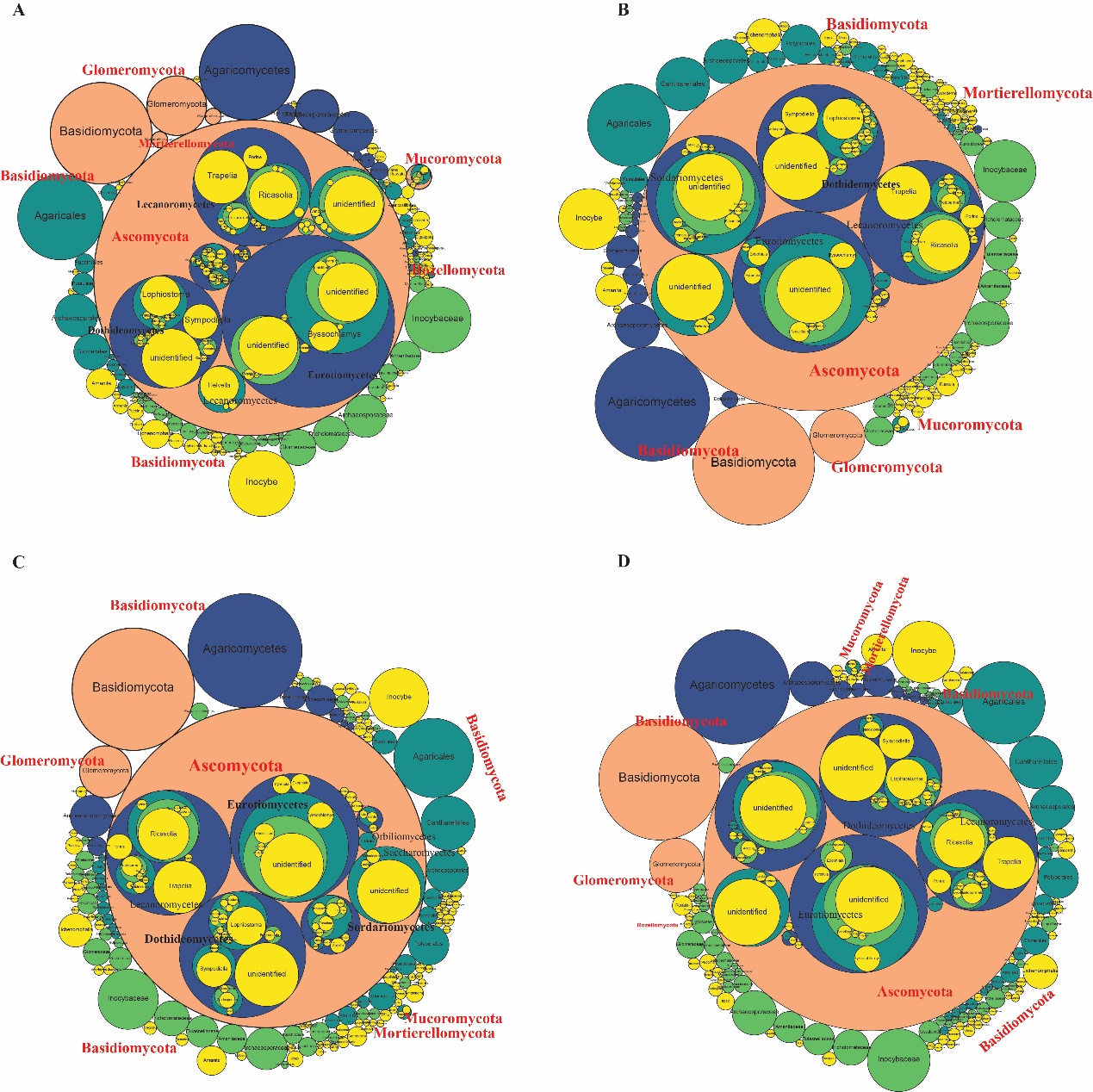


**Figure S3.** Phylum to genus comparative analysis of the microbial composition among seedling generations. **(A-D)** zero, first, third and fifth-generation fungal community compositions. Size of the circles represent their relative abundance and colors showed different taxonomic levels.


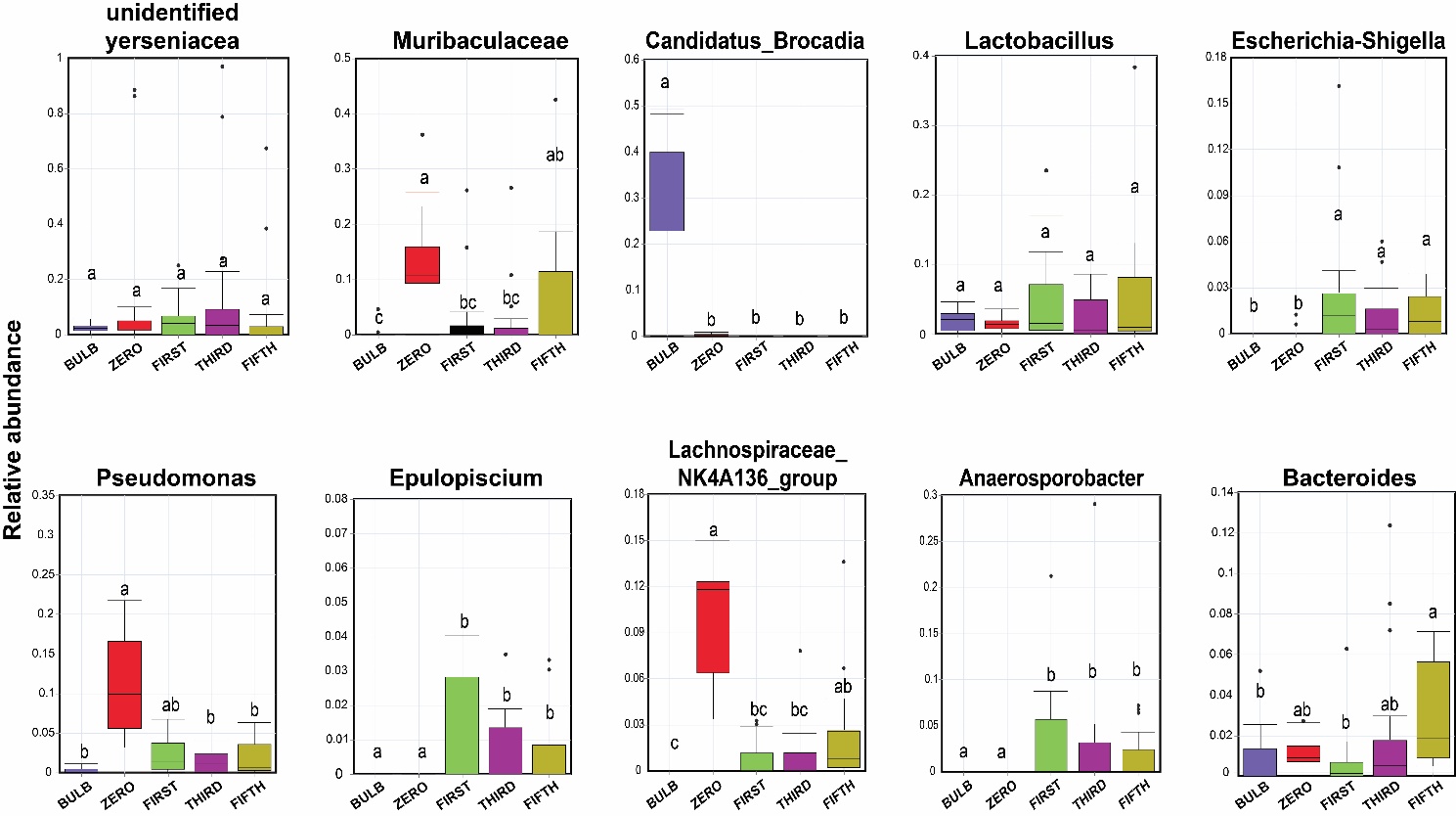


**Figure S4.** The box plots display the significant difference between between differentially abundant bacterial genera. The lowercase letters on each box plot display significant differences among samples (Kruskal wallis test, P < 0.05).


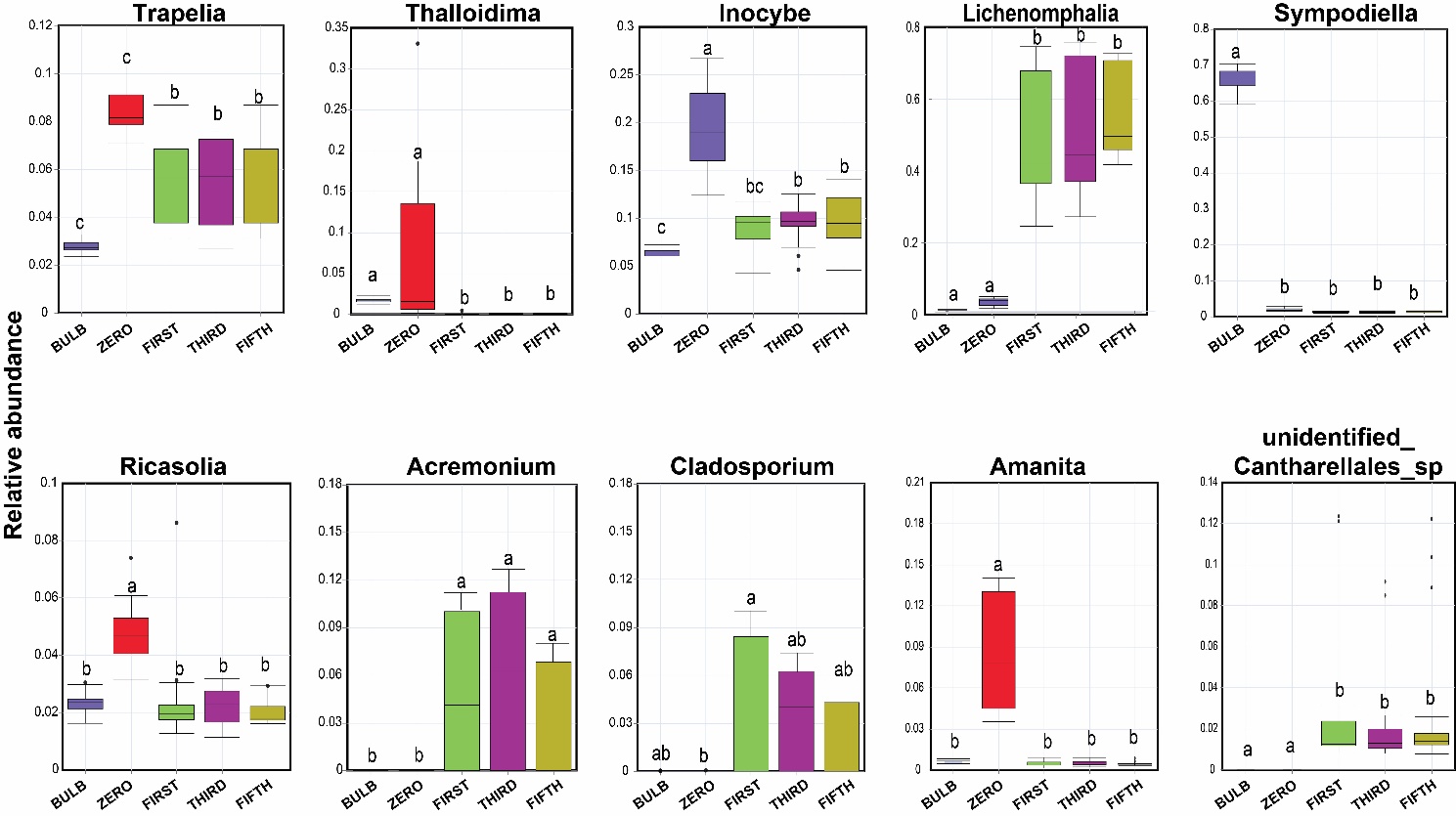


**Figure S5.** The box plots showing the significant difference between differentially abundant fungal genera. The lowercase letters on each box plot display significant differences among samples (Kruskal wallis test, P < 0.05).


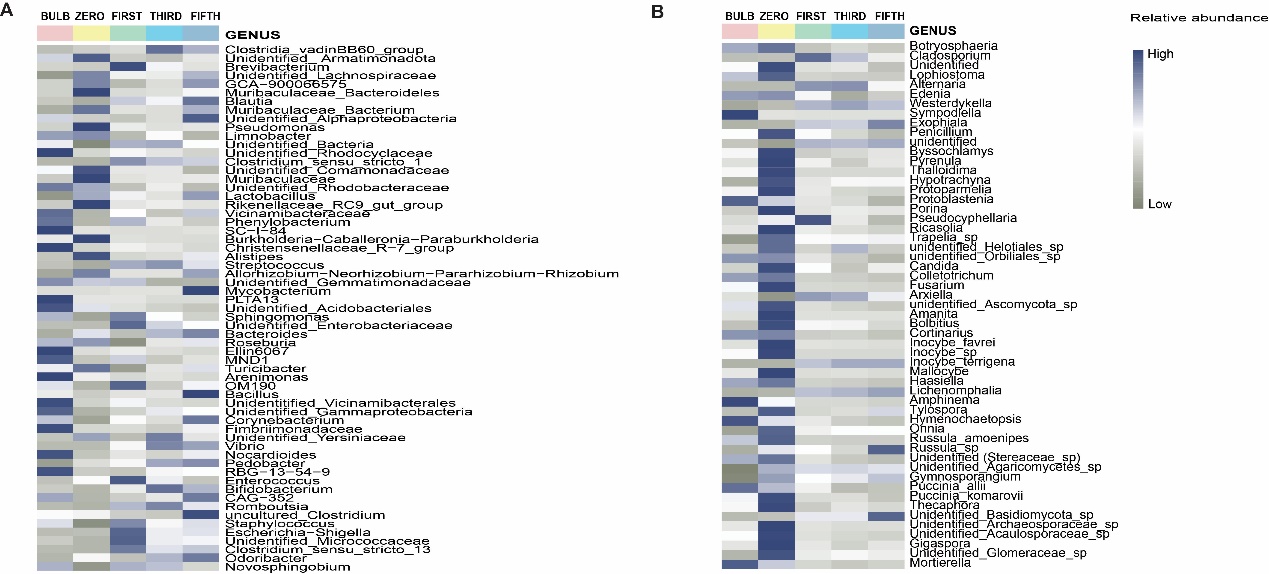


**Figure S6**. Relative abundance of core taxa among different samples **(A)** Bacterial and **(B)** Fungal communities. Heat maps were generated using Bioinformatics.com.cn (https://bioinformatics.com.cn/plot_basic_cluster_heatmap_plot_024). The bluer the color, the higher the relative abundance.


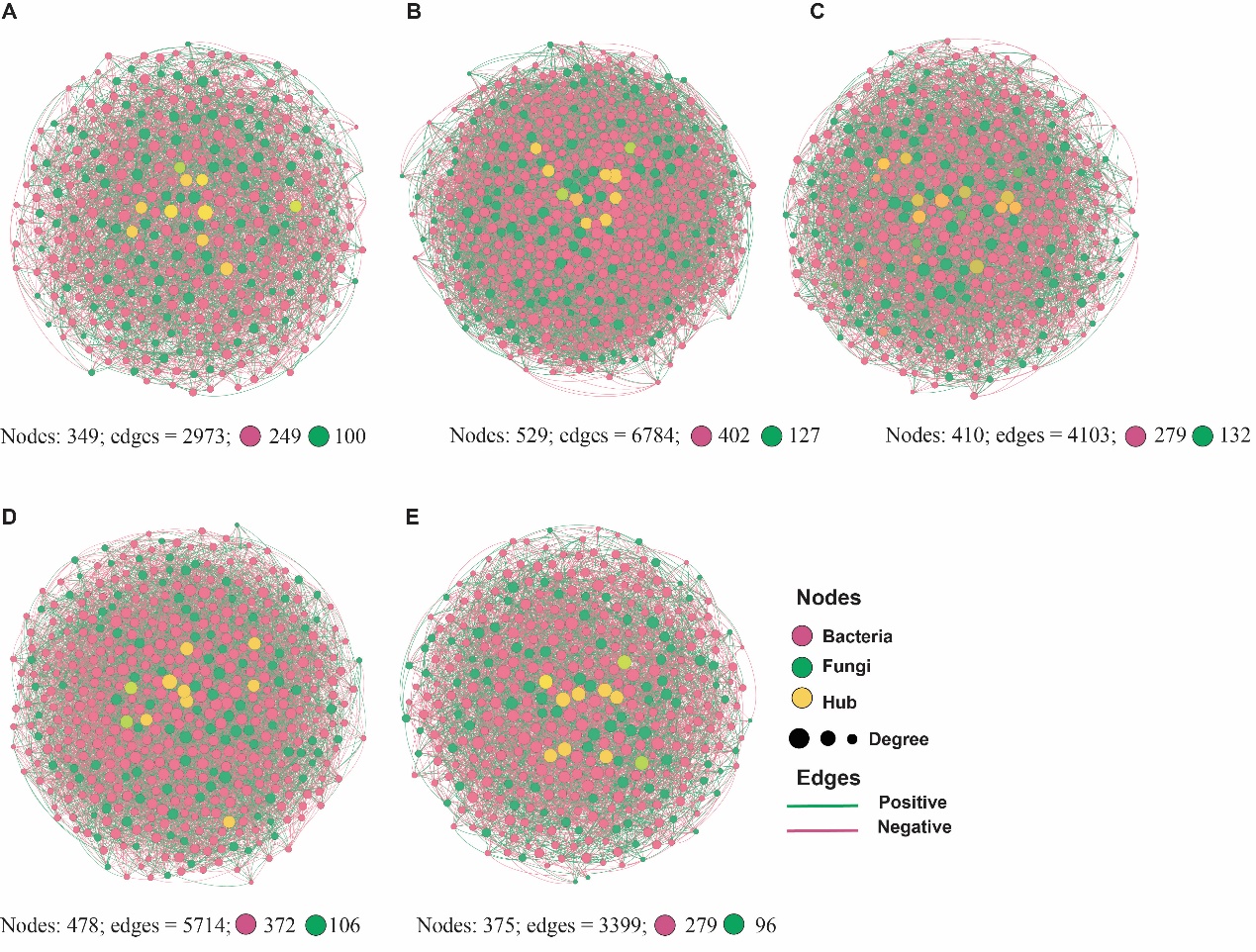


**Figure S7**. Inter-kingdom co-occurrence network analysis at genus level **(A)** Bulb **(B)** Zero generation **(C)** First, **(D)** Third, and **(E)** Fifth generations. Node size indicates the degree of correlation. Pink nodes showed bacterial genera, green indicate fungal genera. The hub nodes is represented by yellow nodes. The green and red lines indicate positive and negative interactions respectively.


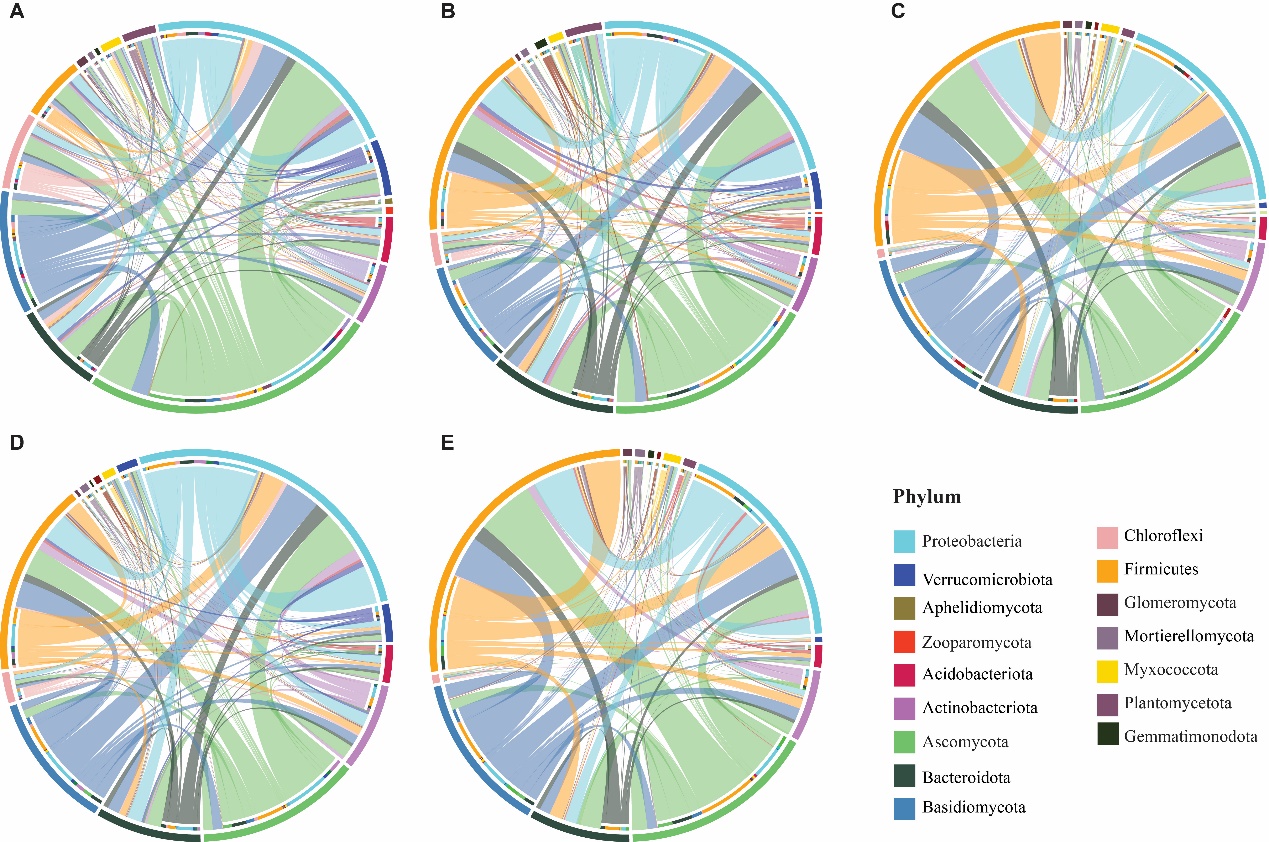


**Figure S8**. Chord plots illustrate the co-occurrence interactions between dominant taxa. Different color indicates different bacterial and fungal phyla.
